# Case Report: Novel Compound Heterozygous Variants in *TRIOBP* Associated With Congenital Deafness in a Chinese Family

**DOI:** 10.3389/fgene.2021.766973

**Published:** 2021-11-17

**Authors:** Cong Zhou, Yuanyuan Xiao, Hanbing Xie, Jing Wang, Shanling Liu

**Affiliations:** ^1^ Department of Obstetrics and Gynecology, West China Second University Hospital, Sichuan University, Chengdu, China; ^2^ Key Laboratory of Birth Defects and Related Diseases of Women and Children, Ministry of Education, Sichuan University, Chengdu, China

**Keywords:** TRIOBP, DFNB28, variation, hearing loss, next generation sequencing

## Abstract

Autosomal recessive non-syndromic deafness-28 (DFNB28) is characterized by prelingual, profound sensorineural hearing loss (HL). The disease is related to variants of the *TRIOBP* gene. TRIO and F-actin binding protein (TRIOBP) plays crucial roles in modulating the assembly of the actin cytoskeleton and are responsible for the proper structure and function of stereocilia in the inner ear. This study aimed to identify pathogenic variants in a patient with HL. Genomic DNA obtained from a 33-year-old woman with HL was evaluated using a disease-targeted gene panel. Using next generation sequencing and bioinformatics analysis, we identified two novel *TRIOBP* c.1170delC (p.S391Pfs*488) and c.3764C > G (p.S1255*) variants. Both parents of the patient were heterozygous carriers of the gene. The two variants have not been reported in general population databases or published literature. The findings of this study will broaden the spectrum of pathogenic variants in the *TRIOBP* gene.

## Introduction

Hearing loss (HL) is one of the most common sensory disorders in humans, affecting 466 million people worldwide. The prevalence of bilateral permanent HL is estimated at one in 900–2,500 newborns, with genetic causes accounting for more than half of the cases (Cynthia and Walter, 2006). Genetic HL can be divided into syndromic and non-syndromic sensorineural HL (SNHL) (Nicolas and Anil, 2002). Hereditary hearing impairment without any other relevant clinical features is referred to as “non-syndromes” and is a genetically heterogeneous disorder (Thomas et al., 2003). To date, 123 non-syndromic HL genes have been identified (http:/hereditaryhearingloss.org/). Nevertheless, pathogenic variants in common HL genes can be identified in only one-third of patients with SNHL (Morag et al., 2018).

Non-syndromic deafness-28 (DFNB28) is related to variants in the *TRIOBP* gene (OMIM: 609761) on chromosome 22q13 (Agnieszka et al., 2017). Multiple isoforms of the protein have been discovered (Beti et al., 2020). Human and mouse *TRIOBP* isoforms are divided into long forms (*TRIOBP*-3, *TRIOBP*-5 and *TRIOBP*-6) and short forms (*TRIOBP*-1, *TRIOBP*-2, and *TRIOBP*-4) (Jian et al., 2015; Jian et al., 2013; Shin et al., 2010). *TRIOBP*-1 consists of a pleckstrin homology (PH) domain near the N-terminal and coiled coil (CC) domains that make up the C-terminal half of the protein. *TRIOBP*-1 is widely expressed and binds to F-actin. It plays an important role in many processes including cell cycle, adhesion, and neuronal differentiation (Beti et al., 2020). *TRIOBP*-2 may encode the N-terminal sections of *TRIOBP*-1 including the PH domain and parts of the CC domain. However, this has not been thoroughly described so far. *TRIOBP*-4 is a 1,144 amino acid protein in humans, which is highly expressed in the hair cells of the inner ear and is crucial for the bundling of actin in the stereocilia of the inner ear, with variants in it causing severe or profound hearing loss (Shin et al., 2010; Beti et al., 2020). *TRIOBP*-5 (also called *TRIOBP*-3 in some earlier articles)encode 2,193 amino acid protein. *TRIOBP*-5 and *TRIOBP*-4 are expressed in the same inner ear cell types, and can be found in static ciliated rootlets. *TRIOBP*-6 is slightly longer than *TRIOBP*-5, which encoding 2,365 amino acid protein. More additional sequences than *TRIOBP*-5 are predicted to be unstructured, except for a stretch of α-helix in the isoform specific N-terminus of *TRIOBP*-6, and another segment near the center of the long isoform. It is likely that the role of *TRIOBP*-6 in the stereocilia may be consistent with *TRIOBP*-5, but this remains unclear because lack of a known murine *TRIOBP*-6 species (Beti et al., 2020; Jian et al., 2015; Jian et al., 2013; Shin et al., 2010).

In the present study, we present a family with isolated, prelingual HL with a recessive inheritance pattern, using 162 targeted genes, a mitochondrial whole gene enrichment panel and Sanger sequencing to identify two novel *TRIOBP* pathogenic variants (c.1170delC, p.S391Pfs*488 and c.3764C > G, p.S1255*) and establish a molecular diagnosis.

### Patient and Methods

#### Patient

**FIGURE 1:**
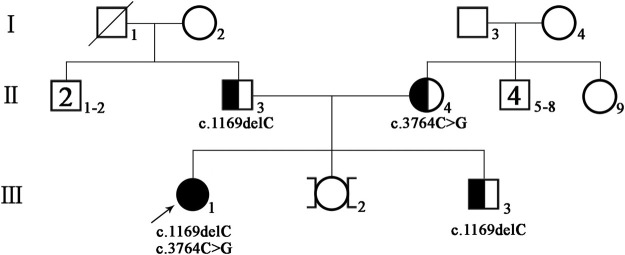
Pedigree of the patient’s family; black-filled shapes represent individuals with hearing loss, and the unfilled shapes represent unaffected ones. Males are represented by squares, females by circles.

A 33-year-old Chinese woman visited the West China Second University Hospital of Sichuan University (Chengdu, China) for genetic diagnosis. The woman’s relatives informed the doctor that the proband had bilateral prelingual deafness and now was trying to conceive. At present, the proband has no other abnormal clinical manifestations except hearing impairment. The proband’s parents are healthy and have no close relative marriage. The mother of the proband denied exposure to teratogenic environmental factors during pregnancy. The pedigree of this family is shown in Figure 1. The present study was approved by the ethics committee of the West China Second University Hospital of Sichuan University, and written informed consent was obtained from the proband and family members.

### DNA Extraction

Peripheral blood samples were drawn from four subjects (Ⅱ3, Ⅱ4, Ⅲ1, and Ⅲ3) after obtaining informed consent. Genomic DNA was extracted from leukocytes of peripheral blood samples using the QIAamp DNA Blood Mini Kit (Qiagen bioinformatics, Hilden, Germany) according to the manufacturer’s instructions. After the DNA was extracted from the samples, the concentration and purity were examined using a NanoDrop 1,000 (Thermo Fisher Scientific, Inc., Wilimington, United States).

### Targeted NGS

According to the manufacturer’s protocol, we used the CM1132 and M113 Kit (MyGenostics, Inc. Beijing, China) to capture and enrich the targeted gDNA of the proband. The CM1132 kit targeted 162 genes (Supplementary Table S1) known to cause deafness, while the M113 kit contained mitochondrial whole genes and hot-spot variants that cause deafness. The double-end sequencing program (PE150) was performed on the NextSeq500 platform (Illumina, Inc. California, United States), and sequence reads of 150bp were received. The reads were mapped to the human genome reference (UCSC GRCh37/hg19) using the Burrows-Wheeler Aligner. Variants were called using the Genome Analysis Tool Kit. Annovar was used to annotate the variants. Then, all the variants were filtered based on their frequency in the 1,000 Genomes Project, ExAC, gnomad, Esp6500. Variants with Minimum allele frequency (MAF) of <0.05 were retained. We then applied several variant prediction tools to predict the functional impact of candidate variants. Finally, the pathogenicities of the variations were analyzed according to the American College of Medical Genetics and Genomics (ACMG) guidelines (Sue et al., 2015) and Expert Specification of the ACMG/AMP variant interpretation guidelines for genetic HL (Andrea et al., 2018). To verify the variations found in the *TRIOBP* gene, Sanger sequencing was performed on samples obtained from the proband and her parents. The variation sites and amplification primers were as follows: c.1170 (forward 5′-CTC​CTC​TCC​CCA​TCG​AAT​CA-3′, reverse 5′-GGT​TCT​GGA​GGC​TTT​GGG​AT-3′) and c.3764 (forward 5′-CTC​CTT​CTC​ATC​CCC​ACC​AC-3′, reverse 5′- TGT​ACT​CCT​CCC​GCT​CCA​A -3′). Sanger sequencing data were analyzed using the Chromas software.

### Variants Detection

**FIGURE 2:**
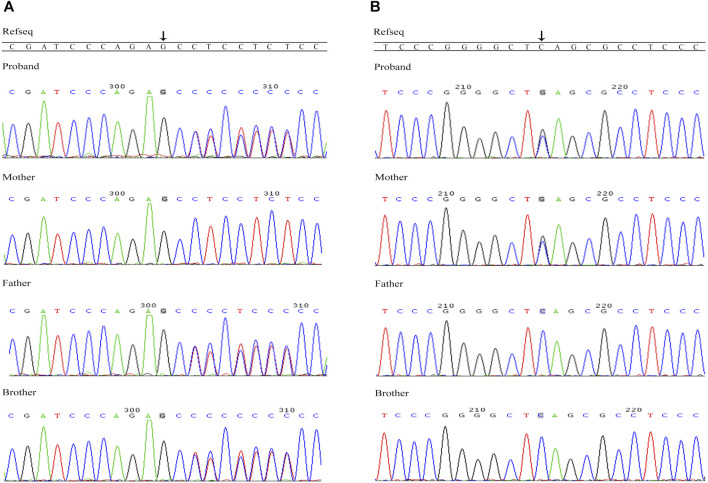
Sanger sequencing confirmation of the variants in *TRIOBP* identified in this study. **(A)** Sequences of the heterozygous frameshift variant c.1170delC (p.S391Pfs*488) and the corresponding wild-type sequence; **(B)** Sequences of the heterozygous Nonsense mutation c.3764C > G (p.S1255*) and the corresponding wild-type sequence.

**FIGURE 3:**
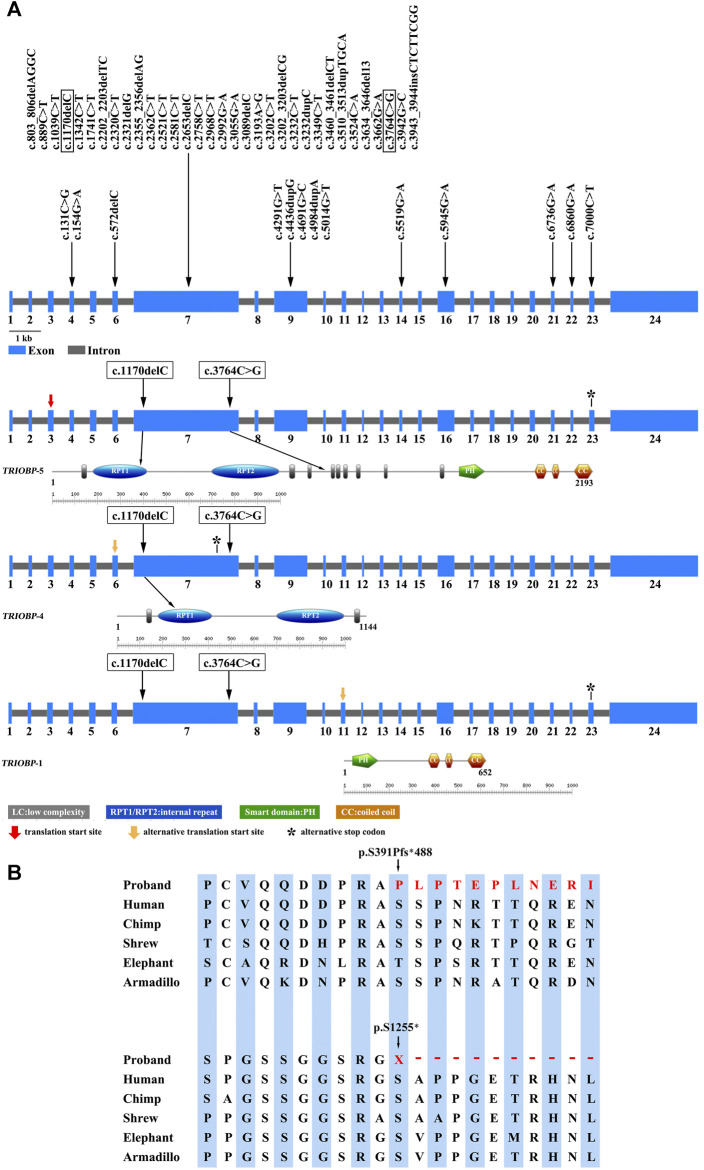
**(A)** Variation spectrum and domain structure of the *TRIOBP* gene (NM_001039141.2). The variations identified in the proband in this study are represented in box. **(B)** Conserved amino acid sequences of TRIOBP (amino acid 391 and 1,255) and the predicted truncated *TRIOBP* caused by the variants identified in this proband.

There were 1,529 variants identified in the targeted regions of the Deafness panel, while no variant related to HL was found in the mitochondrial whole gene panel. The summary of the next generation sequencing (NGS) was listed in Supplementary Table S2). All the 1,529 variants were filtered based on their frequency in the 1,000 Genomes Project, ExAC, gnomad, Esp6500. Variants with MAF of <0.05 were retained. We then applied several variant prediction tools including SIFT, PolyPhen, MutationTaster, GERP and SPIDEX to predict the functional impact of candidate variants. Finally, we founded two variants of the *TRIOBP* (c.1170delC and c.3764C > G, both in exon 7, NM_001039141.2) in the proband. Sanger sequencing of the proband and family members showed that the proband was compound heterozygous, and the parents and the healthy brother were heterozygous carriers (Figures 2A,B). The c.1170delC (p.S391Pfs*488) resulted in a frameshift, while the c.3764C > G (p.S1255*) variant directly introduces a stop codon, both of the variants may lead to TRIOBP truncation. These variants have not been reported in the general population databases, disease databases, or published literature. We analysed the location of the two novel variants in *TRIOBP* and identified that the exon variants were both located in the functional domains (Figure 3A). The positions of the novel variants are highly conserved across several species according to the University of California Santa Cruz Genome Browser database (UCSC) (Figure 3B).

## Discussion and Conclusion

Typically, the pathogenic variants of *TRIOBP* lead to prelingual, severe-to-profound hearing loss. According to the records and descriptions of the relatives, the patient in our study had bilateral prelingual deafness. Unfortunately, she had not received auditory tests at that time. However, not all patients with the *TRIOBP* pathogenic variants showed prelingual deafness, and there were some differences in severity or auditory test results among patients with different variants. According to Agnieszka et al., based on whole exome analysis, they identified two *TRIOBP* pathogenic variants causative of nonsyndromic, peri-to postlingual, moderate-to-severe hearing loss in three siblings from a Polish family (Agnieszka et al., 2017). Differences in age of onset and severity of hearing loss may be related to the location of *TRIOBP* variants and their impact on different isoforms.

DFNB28 is characterized by lingual sensorineural HL and is related to variants in the *TRIOBP.* The *TRIOBP* gene is subject to complicated alternative splicing. There were six splice variants exist, of which three transcripts are most studied, referred to as *TRIOBP*-1, *TRIOBP*-4 and *TRIOBP*-5. Among of them, *TRIOBP*-4 and/or *TRIOBP*-5 are required for hearing (Saima et al., 2006; Nicholas et al., 2014; Tatsuya et al., 2019; Beti et al., 2020). The sequences of *TRIOBP*-5/-4 amino acid contains two repeat motifs, named as the R1 repeat domain (amino acid residues 357–500) and R2 repeat domain (amino acid residues 684–896). The R1 motif is the main actin binding region of *TRIOBP*-4, while the binding of the R2 motif is nonspecific (Jian et al., 2013). *TRIOBP*-5 or *TRIOBP*-1 has a PH and several coiled-coil domains (CCs). There is no overlapping amino acid sequence between *TRIOBP*-1 and *TRIOBP*-4 isoform. The pathogenic variant c.1170delC, p. S391Pfs*488 may disrupts the *TRIOBP*-4 and *TRIOBP*-5 isoforms, whereas the pathogenic variant c.3764C > G, p. S1255* affects only *TRIOBP*-5. Both of the variants without impairing the *TRIOBP*-1 (Figure 3A). The c.1170delC was located in the R1 motifs, and c.3764C > G variants was located in the low complexity The two variants detected in this study were predicted to result in the premature termination of translation (after amino acids 879 and 1,255, respectively). The shortened *TRIOBP* protein are devoid of PH and CC, which is crucial for the actin-binding process (Figure 3A).

*TRIOBP* various is rare in HL patients. There are 45 disease-related variants have been reported in the *TRIOBP* at present (Hashem et al., 2006; Saima et al., 2006; Maiko et al., 2013; Shearer et al., 2014; Christina et al., 2016; Denise et al., 2016; Manou et al., 2016; Agnieszka et al., 2017; Celia et al., 2017; Daniel et al., 2017; Haiqiong et al., 2018; Elodie et al., 2019; Songfeng et al., 2019; Yan et al., 2019; Amal et al., 2020; Birgit et al., 2020; Yongyi et al., 2020), with HL being the only phenotypic manifestation. As presented in Table 1, although the variants cover the region from exon 4 to exon 23, most of the previously reported variations in *TRIOBP* are located in exon 7. The region of exon 7 is defined as a hot point and is more susceptible to variations owing to the accumulation of repeated sequences (Agnieszka et al., 2017). The *TRIOBP* c.1170delC (p.S391Pfs*488) and c.3764C > G (p.S1255*) variants detected in our patient were both located in exon 7. Both variants were classified as pathogenic according to the criteria of ACMG and the Expert Specification of the ACMG/AMP Variant Interpretation Guidelines for Genetic HL (Sue et al., 2015; Andrea et al., 2018).

**TABLE 1:** Disease causing mutations in the *TRIOBP* gene (NM_001039141.2) according to The Human Gene Mutation database (HGMD).

No	Nucleotide change	Protein change	Genomic location (exon)	Mutation type	Phenotype	Ethnicity	Reference
1	c.131C > G	p.S44*	4	Nonsense mutation	Sensorineural hearing loss	Chinese	Yongyi et al. (2020)
2	c.154G > A	p.D52N	4	Missense mutation	Deafness	Japanese	Maiko et al. (2013)
3	c.572delC	p.(Pro191Argfs*50)	6	Frameshift mutation	Deafness, non-syndromic, autosomal recessive	South Africa	Denise et al. (2016)
4	c.803_806	p.(Gln268Leufs*610)	7	Frameshift mutation	Sensorineural hearing loss, postlingual	Polish	Agnieszka et al. (2017)
	delAGGC						
5	c.889C > T	p.Q297*	7	Nonsense mutation	Deafness, non-syndromic	Indian	Saima et al. (2006)
6	c.1039C > T	p.R347*	7	Nonsense mutation	Deafness, non-syndromic	Palestinian	Hashem et al. (2006)
7	c.1342C > T	p.R448*	7	Nonsense mutation	Sensorineural hearing loss	Chinese	Yongyi et al. (2020)
8	c.1741C > T	p.Q581*	7	Nonsense mutation	Deafness, non-syndromic	Palestinian	Hashem et al. (2006)
9	c.2202_2203delTC	p.(Arg735Glnfs*25)	7	Frameshift mutation	Hearing loss	unknown	Amal et al. (2020)
10	c.2320C > T	p.R774*	7	Nonsense mutation	Deafness autosomal recessive 28	unknown	Daniel et al. (2017)
11	c.2321delG	p.(Arg774Hisfs*105)	7	Frameshift mutation	Hearing loss	Chinese	Yan et al. (2019)
12	c.2355_2356delAG	p.(Arg785Serfs*50)	7	Frameshift mutation	Hearing loss, non-syndromic, autosomal recessive	Turkish	Agnieszka et al. (2017)
13	c.2362C > T	p.R788*	7	Nonsense mutation	Deafness, non-syndromic	Pakistani	Saima et al. (2006)
14	c.2521C > T	p.R841*	7	Nonsense mutation	Deafness, non-syndromic, autosomal recessive	Turkey	Denise et al. (2016)
15	c.2581C > T	p.R861*	7	Nonsense mutation	Hearing loss, non-syndromic, autosomal recessive	Chinese	Haiqiong et al. (2018)
16	c.2653delC	p.(Arg885Alafs*120)	7	Frameshift mutation	Hearing impairment	Dutch	Celia et al. (2017)
17	c.2758C > T	p.R920*	7	Nonsense mutation	Hearing loss, non-syndromic, autosomal recessive	Chinese	Haiqiong et al. (2018)
18	c.2968C > T	p.R990*	7	Nonsense mutation	Hearing loss	Iran	Haiqiong et al. (2018)
19	c.2992G > A	p.A998T	7	Missense mutation	Hearing loss	unknown	Christina et al. (2016)
20	c.3055G > A	p.G1019R	7	Missense mutation	Deafness, non-syndromic	Palestinian	Hashem et al. (2006)
21	c.3089delC	p.(Pro1030Leufs*183)	7	Frameshift mutation	Deafness, non-syndromic, autosomal recessive	United States of America	Denise et al. (2016)
22	c.3193A > G	p.I1065V	7	Missense mutation	Deafness, autosomal recessive	Chinese	Songfeng et al. (2019)
23	c.3202C > T	p.R1068*	7	Nonsense mutation	Deafness, non-syndromic	Pakistani	Saima et al. (2006)
24	c.3202_3203delCG	p.(Asp1069Cysfs*14)	7	Frameshift mutation	Deafness, non-syndromic	Indian	Saima et al. (2006)
25	c.3232C > T	p.R1078C	7	Missense mutation	Hearing loss, non-syndromic, autosomal recessive	Western-European	Manou et al. (2016)
26	c.3232dupC	p.(Arg1078Profs*6)	7	Frameshift mutation	Deafness, non-syndromic	Indian	Saima et al. (2006)
27	c.3349C > T	p.R1117*	7	Nonsense mutation	Deafness, non-syndromic	Indian	Saima et al. (2006)
28	c.3460_3461delCT	p.(Leu1154Alafs*29)	7	Frameshift mutation	Hearing impairment	Dutch	Celia et al. (2017)
29	c.3510_3513dupTGCA	p.(Pro1172Cysfs*13)	7	Frameshift mutation	Deafness, non-syndromic, autosomal recessive	South Africa	Denise et al. (2016)
30	c.3524C > A	p.S1175*	7	Nonsense mutation	Sensorineural hearing loss	Chinese	Yongyi et al. (2020)
31	c.3634_3646del13	p.(Leu1212Cysfs*22)	7	Frameshift mutation	Hearing loss, non-syndromic	Pakistani	Elodie et al. (2019)
32	c.3662G > A	p.R1221Q	7	Missense mutation	Hearing loss	unknown	Manou et al. (2016)
33	c.3942G > C	p.E1314D	7	Missense mutation	Hearing loss	unknown	Manou et al. (2016)
34	c.3943_3944insCTCTTCGG	p.(Arg1315Profs*44)	7	Frameshift mutation	Hearing loss	Chinese	Yan et al. (2019)
35	c.4291G > T	p.E1431*	9	Nonsense mutation	Sensorineural hearing loss	Chinese	Yongyi et al. (2020)
36	c.4436dupG	p.(Thr1480Hisfs*22)	9	Frameshift mutation	Hearing loss	Chinese	Yan et al. (2019)
37	c.4691G > C	p.G1564A	9	Missense mutation	Hearing loss	unknown	Christina et al. (2016)
38	c.4984dupA	p.(Thr1662Asnfs*48)	9	Frameshift mutation	Hearing loss	Egypt	Birgit et al. (2020)
39	c.5014G > T	p.G1672*	9	Nonsense mutation	Hearing impairment	Polish/Dutch	Celia et al. (2017)
40	c.5519G > A	p.R1840H	14	Missense mutation	Deafness	Japanese	Maiko et al. (2013)
41	c.5945G > A	p.R1982H	16	Missense mutation	Deafness, autosomal recessive	Chinese	Songfeng et al. (2019)
42	c.6736G > A	p.E2246K	21	Missense mutation	Hearing loss	unknown	Christina et al. (2016)
43	c.6860G > A	p.R2287H	22	Missense mutation	Deafness	Japanese	Maiko et al. (2013)
44	c.7000C > T	p.R2334W	23	Missense mutation	Hearing loss, non-syndromic, autosomal recessive	Western-European	Manou et al. (2016)
45	Not yet available	Not yet available	Not yet available	Gross deletions	Hearing loss, non-syndromic	unknown	Shearer et al. (2014)

This report describes a Chinese patient with a hearing impairment. Using disease-targeted gene panels, we identified two novel compound heterozygous variants in the *TRIOBP* gene. Both variants were predicted to lead to premature termination codons, resulting in a truncated *TRIOBP* protein formation. The two novel *TRIOBP* variants expand the spectrum of *TRIOBP* variants in HL. Although *TRIOBP* variants are not a frequent cause of HL, this gene should be thoroughly analyzed in patients with prelingua HL.

## Data Availability

The raw datasets analysed during the current study are not deposited in publicly available repositories because of considerations about the security of human genetic resources and patient anonymity, but are available from the corresponding author on reasonable request.

## Abbreviations

DFNB28, The autosomal recessive non-syndromic deafness-28; HL, Hearing Loss; SNHL, sensorineural hearing loss; Genomic(g) DNA; ACMG, American college of medical genetics and genomics; NGS, next generation sequencing; UCSC, university of California santa cruz genome browser database; PH, pleckstrin homology domain; CC, coiled-coil domains.
