# Supplementary figures and images for: Case Report: Novel Compound Heterozygous Variants in TRIOBP Associated With Congenital Deafness in a Chinese Family
Source: Front Genet. 2021 Nov 17;12:766973. doi: 10.3389/fgene.2021.766973 (PMC8635749; doi:10.3389/fgene.2021.766973)

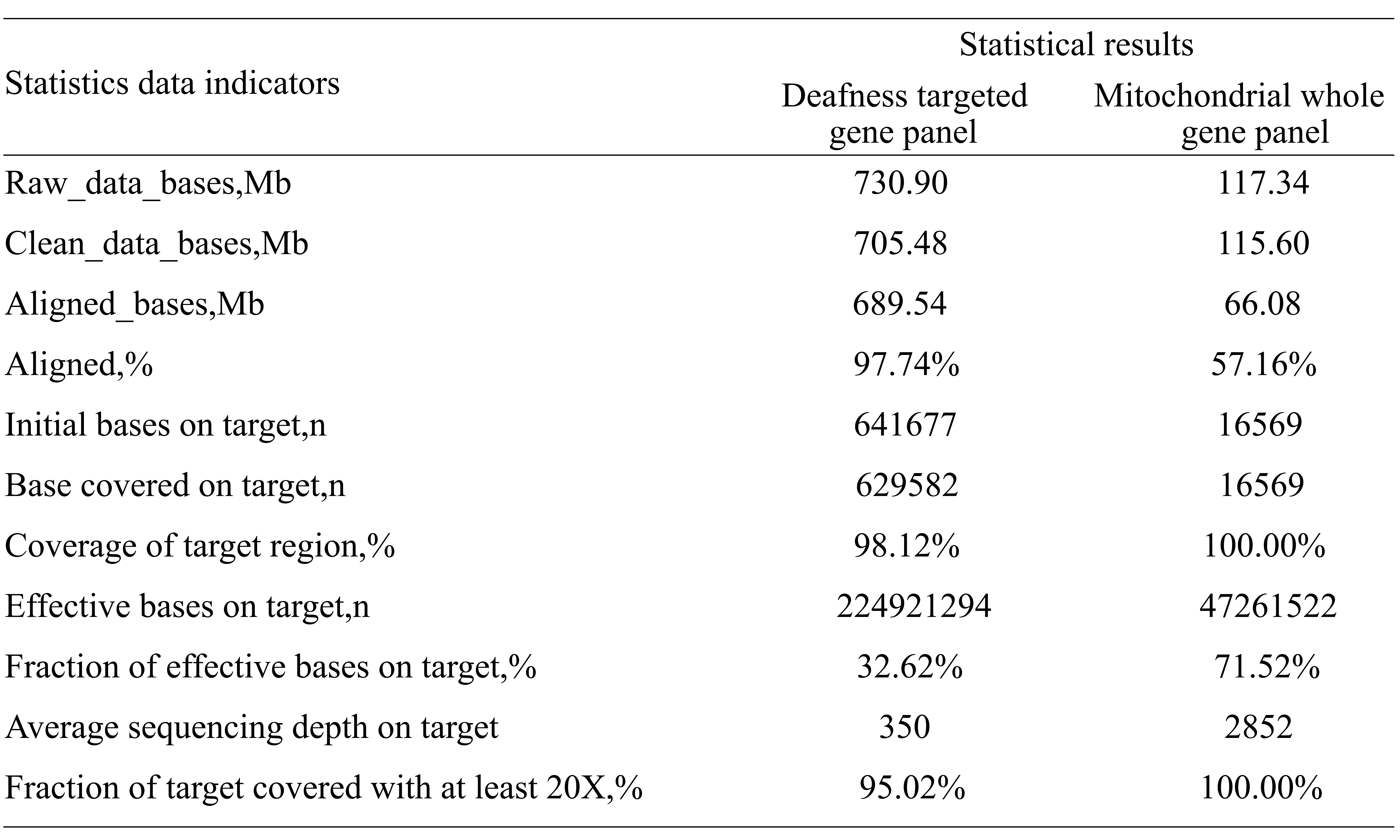

Supplement: Supplementary file 1 [file Image2.JPEG]

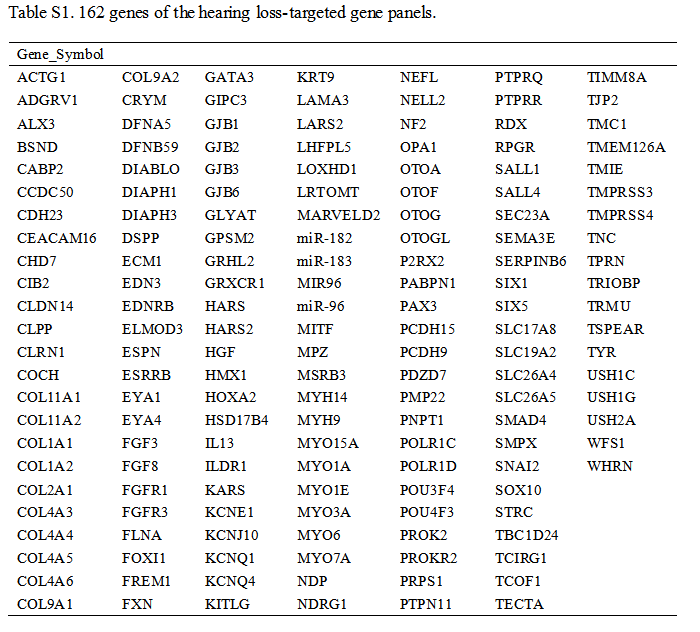

Supplement: Supplementary file 2 [file Image1.PNG]
